# *catena*-Poly[[diaqua­magnesium(II)]-bis­(μ-5-ammonio­isophthalato-κ^2^ *O* ^1^:*O* ^3^)]

**DOI:** 10.1107/S1600536810040250

**Published:** 2010-10-23

**Authors:** Cheng-You Wu, Chia-Her Lin

**Affiliations:** a Department of Chemistry, Chung-Yuan Christian University, Chung-Li 320, Taiwan

## Abstract

In the title compound, [Mg(C_8_H_6_NO_4_)_2_(H_2_O)_2_]_*n*_, the Mg^II^ ion lies on a twofold roatation axis and is coordinated in a slightly distorted octa­hedral environment. Pairs of bridging ammonium­isophthalate ligands connect symmetry-related Mg^II^ ions, forming chains along [010]. In the crystal, inter­molecular O—H⋯O and N—H⋯O hydrogen bonds link these chains into a three-dimensional network. The centroids of pairs of symmetry-related benzene rings within a chain are separated by 3.5707 (12) Å.

## Related literature

For general background to metal coordination polymers, see: Kitagawa *et al.* (2004 ▶). For related structures, see: Zeng *et al.* (2007 ▶); Kongshaug & Fjellvåg (2006 ▶).
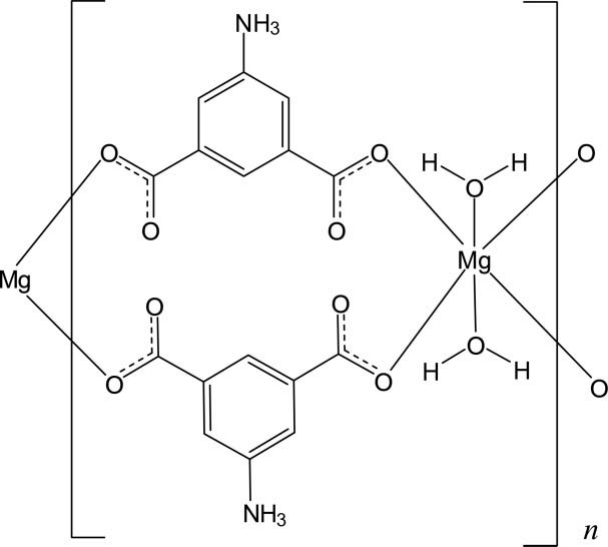


## Experimental

### 

#### Crystal data

[Mg(C_8_H_6_NO_4_)_2_(H_2_O)_2_]
*M* *_r_* = 420.62
Monoclinic, 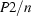

*a* = 6.9987 (2) Å
*b* = 9.9434 (3) Å
*c* = 11.3809 (3) Å
β = 94.730 (2)°
*V* = 789.31 (4) Å^3^
*Z* = 2
Mo *K*α radiation
μ = 0.18 mm^−1^
*T* = 295 K
0.10 × 0.08 × 0.08 mm

#### Data collection

Bruker APEXII CCD diffractometer
Absorption correction: multi-scan (*SADABS*; Bruker, 2008 ▶) *T*_min_ = 0.982, *T*_max_ = 0.986
6693 measured reflections
1963 independent reflections
1228 reflections with *I* > 2σ(*I*)
*R*_int_ = 0.047

#### Refinement

*R*[*F* ^2^ > 2σ(*F* ^2^)] = 0.049
*wR*(*F* ^2^) = 0.136
*S* = 1.00
1963 reflections
132 parameters
2 restraints
H-atom parameters constrained
Δρ_max_ = 0.36 e Å^−3^
Δρ_min_ = −0.29 e Å^−3^

### 

Data collection: *APEX2* (Bruker, 2010 ▶); cell refinement: *SAINT* (Bruker, 2009 ▶); data reduction: *SAINT*; program(s) used to solve structure: *SHELXS97* (Sheldrick, 2008 ▶); program(s) used to refine structure: *SHELXL97* (Sheldrick, 2008 ▶); molecular graphics: *DIAMOND* (Brandenburg, 2010 ▶); software used to prepare material for publication: *SHELXTL* (Sheldrick, 2008 ▶).

## Supplementary Material

Crystal structure: contains datablocks I, global. DOI: 10.1107/S1600536810040250/lh5144sup1.cif

Structure factors: contains datablocks I. DOI: 10.1107/S1600536810040250/lh5144Isup2.hkl

Additional supplementary materials:  crystallographic information; 3D view; checkCIF report

## Figures and Tables

**Table 1:** Hydrogen-bond geometry (Å, °)

*D*—H⋯*A*	*D*—H	H⋯*A*	*D*⋯*A*	*D*—H⋯*A*
O1*W*—H1*WA*⋯O3^i^	0.85	2.04	2.883 (2)	175
N1—H1*A*⋯O1^ii^	0.89	1.85	2.726 (2)	166
N1—H1*B*⋯O2^iii^	0.89	2.19	2.919 (3)	138
N1—H1*B*⋯O4^iv^	0.89	2.26	3.009 (3)	142
N1—H1*C*⋯O3^v^	0.89	2.00	2.869 (2)	165

Symmetry codes: (i) 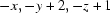; (ii) 
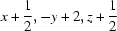
; (iii) 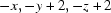; (iv) 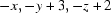; (v) 
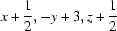
.

**Fig. 1.**
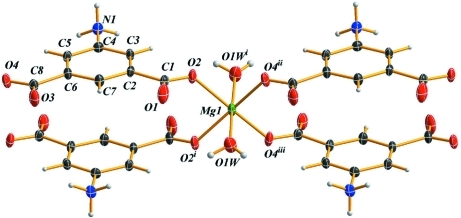
Part of the one-dimensional chain title compound with labelling and displacement ellipsoids drawn at the 50% probability level. Symmetry codes: (i) -x + 1/2, y, -z + 3/2; (ii) x, y - 1, z; (iii) -x + 1/2, y - 1, -z + 3/2.

**Fig. 2.**
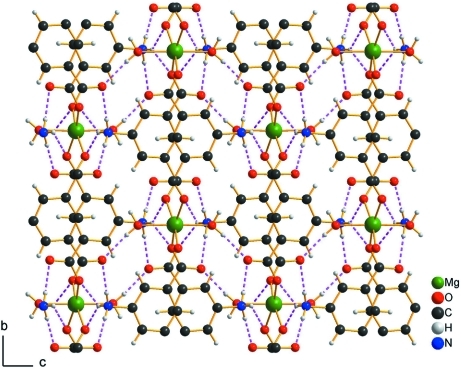
Part of the crystal structure of the title compound with view along the crystallographic a axis with hydrogen bonds shown as dashed lines.

## supplementary crystallographic information

### Comment

The synthesis of metal coordination polymers has been an intense research area due to their interesting topologies and potential applications (Kitagawa, *et al.*, 2004). The crystal structures of 5-aminoisophthalic acid complexes with sodium (Zeng, *et al.*, 2007) and zinc (Kongshaug, *et al.*, 2006) have already been reported. In our continuous investigation in this area we report herein the structure of a new Mg coordination polymer based on the 5-amineisophthalato ligand.

The asymmetric unit of the title compound consists of half a an Mg^II^ ion, one 5-ammoniumisophthalato ligand and one coordinated water molecule. The Mg^II^ ion lies on a twofold roatation axis and is coordinated in a slightly distorted octahedral coordination environment (see Fig. 1). Pairs of bridging ammoniumisophthalato ligands connect symmetry related Mg^II^ ions to form one-dimensional chains along [010]. In the crystal structure, intermolecular O-H···O and N-H···O hydrogen bonds link these chains into a three-dimensional network (Fig. 2). The centroids of pairs of symmetry related benzene rings within a chain are separated by 3.5707 (12)Å.

### Experimental

Solvothermal reactions were carried out at 423 K for 2 d in a Teflon-lined acid digestion bomb with an internal volume of 23 ml followed by slow cooling at 6 K/h to room temperature. A single-phase product consisting of transparent brown crystals of was obtained from a mixture of 5-aminoisophthalic acid (C_8_H_7_NO_4_, 0.0724 g, 0.4 mmol), Mg(NO_3_)_2_.6H_2_O (0.1026 g, 0.4 mmol), and DMF (5.0 ml) and H_2_O (1.0 ml).

### Refinement

H atoms were constrained to ideal geometries, with C—H = 0.93 Å and *U*_iso_(H) = 1.2*U*_eq_(C); O—H = 0.85 Å and *U*_iso_(H) = 1.5*U*_eq_(N); N—H = 0.89 Å and *U*_iso_(H) = 1.5*U*_eq_(N). The aqua H atoms are clearly visible in difference Fourier maps and this clarifies that one of the H atoms does not have an acceptor.

### Crystal data

**Table:**

[Mg(C_8_H_6_NO_4_)_2_(H_2_O)_2_]	*F*(000) = 436
*M**_r_* = 420.62	*D*_x_ = 1.770 Mg m^−^^3^
Monoclinic, *P*2/*n*	Mo *K*α radiation, λ = 0.71073 Å
Hall symbol: -P 2yac	Cell parameters from 1773 reflections
*a* = 6.9987 (2) Å	θ = 2.7–28.1°
*b* = 9.9434 (3) Å	µ = 0.18 mm^−^^1^
*c* = 11.3809 (3) Å	*T* = 295 K
β = 94.730 (2)°	Columnar, colourless
*V* = 789.31 (4) Å^3^	0.10 × 0.08 × 0.08 mm
*Z* = 2	

### Data collection

**Table:**

Bruker APEXII CCD diffractometer	1963 independent reflections
Radiation source: fine-focus sealed tube	1228 reflections with *I* > 2σ(*I*)
graphite	*R*_int_ = 0.047
Detector resolution: 8.3333 pixels mm^-1^	θ_max_ = 28.3°, θ_min_ = 2.1°
φ and ω scans	*h* = −9→9
Absorption correction: multi-scan (*SADABS*; Bruker, 2008)	*k* = −13→12
*T*_min_ = 0.982, *T*_max_ = 0.986	*l* = −15→15
6693 measured reflections	

### Refinement

**Table:**

Refinement on *F*^2^	Primary atom site location: structure-invariant direct methods
Least-squares matrix: full	Secondary atom site location: difference Fourier map
*R*[*F*^2^ > 2σ(*F*^2^)] = 0.049	Hydrogen site location: inferred from neighbouring sites
*wR*(*F*^2^) = 0.136	H-atom parameters constrained
*S* = 1.00	*w* = 1/[σ^2^(*F*_o_^2^) + (0.0686*P*)^2^] where *P* = (*F*_o_^2^ + 2*F*_c_^2^)/3
1963 reflections	(Δ/σ)_max_ < 0.001
132 parameters	Δρ_max_ = 0.36 e Å^−^^3^
2 restraints	Δρ_min_ = −0.28 e Å^−^^3^

### Special details

**Table:**

Geometry. All e.s.d.'s (except the e.s.d. in the dihedral angle between two l.s. planes) are estimated using the full covariance matrix. The cell e.s.d.'s are taken into account individually in the estimation of e.s.d.'s in distances, angles and torsion angles; correlations between e.s.d.'s in cell parameters are only used when they are defined by crystal symmetry. An approximate (isotropic) treatment of cell e.s.d.'s is used for estimating e.s.d.'s involving l.s. planes.
Refinement. Refinement of *F*^2^ against ALL reflections. The weighted *R*-factor *wR* and goodness of fit *S* are based on *F*^2^, conventional *R*-factors *R* are based on *F*, with *F* set to zero for negative *F*^2^. The threshold expression of *F*^2^ > σ(*F*^2^) is used only for calculating *R*-factors(gt) *etc*. and is not relevant to the choice of reflections for refinement. *R*-factors based on *F*^2^ are statistically about twice as large as those based on *F*, and *R*- factors based on ALL data will be even larger.

### Fractional atomic coordinates and isotropic or equivalent isotropic displacement parameters (Å^2^)

**Table:**

	*x*	*y*	*z*	*U*_iso_*/*U*_eq_	
Mg1	0.2500	0.72884 (11)	0.7500	0.0186 (3)	
O1	−0.0895 (3)	0.97847 (17)	0.64235 (14)	0.0356 (5)	
O2	0.0658 (2)	0.87721 (15)	0.79595 (13)	0.0242 (4)	
O3	−0.1076 (3)	1.47494 (16)	0.61801 (14)	0.0294 (5)	
O4	0.0357 (2)	1.59288 (15)	0.76552 (13)	0.0240 (4)	
C1	−0.0008 (4)	0.9804 (2)	0.74109 (19)	0.0192 (5)	
C2	0.0340 (3)	1.1145 (2)	0.80116 (18)	0.0165 (5)	
C3	0.1175 (3)	1.1241 (2)	0.91552 (18)	0.0175 (5)	
H3A	0.1514	1.0469	0.9585	0.021*	
C4	0.1497 (3)	1.2498 (2)	0.96473 (18)	0.0164 (5)	
C5	0.1056 (3)	1.3660 (2)	0.90294 (18)	0.0173 (5)	
H5A	0.1308	1.4495	0.9376	0.021*	
C6	0.0226 (3)	1.3570 (2)	0.78786 (18)	0.0169 (5)	
C7	−0.0154 (3)	1.2315 (2)	0.73840 (19)	0.0177 (5)	
H7A	−0.0746	1.2253	0.6624	0.021*	
C8	−0.0199 (3)	1.4837 (2)	0.71758 (18)	0.0186 (5)	
O1W	0.1775 (3)	0.72574 (17)	0.56326 (14)	0.0317 (5)	
H1WA	0.1547	0.6707	0.5072	0.048*	
H1WB	0.1906	0.8003	0.5266	0.048*	
N1	0.2340 (3)	1.26019 (18)	1.08656 (15)	0.0203 (5)	
H1A	0.3092	1.1894	1.1034	0.030*	
H1B	0.1409	1.2622	1.1353	0.030*	
H1C	0.3031	1.3352	1.0949	0.030*	

### Atomic displacement parameters (Å^2^)

**Table:**

	*U*^11^	*U*^22^	*U*^33^	*U*^12^	*U*^13^	*U*^23^
Mg1	0.0252 (7)	0.0106 (5)	0.0192 (5)	0.000	−0.0031 (5)	0.000
O1	0.0543 (14)	0.0189 (10)	0.0303 (10)	−0.0015 (9)	−0.0166 (9)	−0.0080 (7)
O2	0.0343 (11)	0.0106 (8)	0.0274 (9)	0.0030 (7)	0.0011 (8)	−0.0002 (6)
O3	0.0446 (13)	0.0171 (9)	0.0245 (8)	0.0021 (8)	−0.0098 (8)	0.0042 (7)
O4	0.0346 (11)	0.0114 (8)	0.0253 (8)	−0.0057 (7)	−0.0013 (8)	0.0006 (6)
C1	0.0233 (14)	0.0113 (11)	0.0227 (11)	−0.0023 (10)	−0.0008 (10)	−0.0037 (9)
C2	0.0190 (13)	0.0094 (11)	0.0209 (11)	0.0001 (9)	0.0009 (10)	−0.0015 (8)
C3	0.0241 (14)	0.0109 (11)	0.0173 (10)	0.0005 (9)	0.0009 (10)	0.0018 (8)
C4	0.0171 (12)	0.0174 (12)	0.0142 (10)	−0.0007 (9)	−0.0014 (9)	−0.0010 (8)
C5	0.0228 (14)	0.0115 (11)	0.0175 (10)	−0.0007 (9)	0.0004 (10)	−0.0031 (8)
C6	0.0183 (13)	0.0114 (11)	0.0206 (11)	0.0009 (9)	−0.0010 (10)	0.0021 (8)
C7	0.0209 (13)	0.0146 (11)	0.0167 (10)	−0.0012 (10)	−0.0044 (9)	0.0002 (8)
C8	0.0236 (14)	0.0137 (12)	0.0183 (11)	0.0018 (10)	0.0001 (10)	0.0032 (8)
O1W	0.0477 (13)	0.0250 (10)	0.0211 (8)	−0.0054 (9)	−0.0047 (8)	−0.0004 (7)
N1	0.0260 (12)	0.0177 (10)	0.0162 (9)	0.0003 (8)	−0.0043 (8)	−0.0009 (7)

### Geometric parameters (Å, °)

**Table:**

Mg1—O4^i^	2.0375 (17)	C3—C4	1.380 (3)
Mg1—O4^ii^	2.0375 (17)	C3—H3A	0.9300
Mg1—O2	2.0550 (17)	C4—C5	1.375 (3)
Mg1—O2^iii^	2.0550 (17)	C4—N1	1.465 (3)
Mg1—O1W	2.1441 (16)	C5—C6	1.391 (3)
Mg1—O1W^iii^	2.1441 (16)	C5—H5A	0.9300
O1—C1	1.238 (3)	C6—C7	1.386 (3)
O2—C1	1.270 (3)	C6—C8	1.509 (3)
O3—C8	1.246 (3)	C7—H7A	0.9300
O4—C8	1.262 (3)	O1W—H1WA	0.8459
O4—Mg1^iv^	2.0374 (17)	O1W—H1WB	0.8589
C1—C2	1.508 (3)	N1—H1A	0.8900
C2—C3	1.385 (3)	N1—H1B	0.8900
C2—C7	1.394 (3)	N1—H1C	0.8900
			
O4^i^—Mg1—O4^ii^	96.86 (11)	C2—C3—H3A	120.5
O4^i^—Mg1—O2	88.43 (7)	C5—C4—C3	122.1 (2)
O4^ii^—Mg1—O2	168.54 (7)	C5—C4—N1	118.73 (19)
O4^i^—Mg1—O2^iii^	168.54 (7)	C3—C4—N1	119.18 (19)
O4^ii^—Mg1—O2^iii^	88.43 (7)	C4—C5—C6	119.1 (2)
O2—Mg1—O2^iii^	88.24 (10)	C4—C5—H5A	120.4
O4^i^—Mg1—O1W	87.75 (7)	C6—C5—H5A	120.4
O4^ii^—Mg1—O1W	91.16 (7)	C7—C6—C5	119.4 (2)
O2—Mg1—O1W	99.23 (7)	C7—C6—C8	120.9 (2)
O2^iii^—Mg1—O1W	81.96 (7)	C5—C6—C8	119.6 (2)
O4^i^—Mg1—O1W^iii^	91.16 (7)	C6—C7—C2	120.8 (2)
O4^ii^—Mg1—O1W^iii^	87.75 (7)	C6—C7—H7A	119.6
O2—Mg1—O1W^iii^	81.96 (7)	C2—C7—H7A	119.6
O2^iii^—Mg1—O1W^iii^	99.23 (7)	O3—C8—O4	124.4 (2)
O1W—Mg1—O1W^iii^	178.35 (11)	O3—C8—C6	119.0 (2)
C1—O2—Mg1	131.89 (15)	O4—C8—C6	116.66 (19)
C8—O4—Mg1^iv^	137.42 (15)	Mg1—O1W—H1WA	140.5
O1—C1—O2	124.7 (2)	Mg1—O1W—H1WB	116.3
O1—C1—C2	118.4 (2)	H1WA—O1W—H1WB	102.3
O2—C1—C2	116.89 (19)	C4—N1—H1A	109.5
C3—C2—C7	119.4 (2)	C4—N1—H1B	109.5
C3—C2—C1	121.77 (19)	H1A—N1—H1B	109.5
C7—C2—C1	118.78 (19)	C4—N1—H1C	109.5
C4—C3—C2	119.06 (19)	H1A—N1—H1C	109.5
C4—C3—H3A	120.5	H1B—N1—H1C	109.5

Symmetry codes: (i) *x*, *y*−1, *z*; (ii) −*x*+1/2, *y*−1, −*z*+3/2; (iii) −*x*+1/2, *y*, −*z*+3/2; (iv) *x*, *y*+1, *z*.

### Hydrogen-bond geometry (Å, °)

**Table:**

*D*—H···*A*	*D*—H	H···*A*	*D*···*A*	*D*—H···*A*
O1W—H1WA···O3^v^	0.85	2.04	2.883 (2)	175
N1—H1A···O1^vi^	0.89	1.85	2.726 (2)	166
N1—H1B···O2^vii^	0.89	2.19	2.919 (3)	138
N1—H1B···O4^viii^	0.89	2.26	3.009 (3)	142
N1—H1C···O3^ix^	0.89	2.00	2.869 (2)	165

Symmetry codes: (v) −*x*, −*y*+2, −*z*+1; (vi) *x*+1/2, −*y*+2, *z*+1/2; (vii) −*x*, −*y*+2, −*z*+2; (viii) −*x*, −*y*+3, −*z*+2; (ix) *x*+1/2, −*y*+3, *z*+1/2.
